# Greenhouse gas emissions from synthetic nitrogen manufacture and fertilization for main upland crops in China

**DOI:** 10.1186/s13021-019-0133-9

**Published:** 2019-12-30

**Authors:** Rushan Chai, Xinxin Ye, Chao Ma, Qingyun Wang, Renfeng Tu, Ligan Zhang, Hongjian Gao

**Affiliations:** Anhui Province Key Lab of Farmland Ecological Conservation and Pollution Prevention, School of Resources and Environment, Anhui Agricultural University, Hefei, China

**Keywords:** Wheat, Maize, Synthetic N fertilizers, Manufacture, Application, Greenhouse gas emissions

## Abstract

**Background:**

A significant source of greenhouse gas (GHG) emissions comes from the manufacture of synthetic nitrogen (N) fertilizers consumed in crop production processes. And the application of synthetic N fertilizers is recognized as the most important factor contributing to direct N_2_O emissions from agricultural soils. Based on statistical data and relevant literature, the GHG emissions associated with synthetic N manufacture and fertilization for wheat and maize in different provinces and agricultural regions of China were quantitatively evaluated in the present study.

**Results:**

During the 2015–2017 period, the average application rates of synthetic N for wheat and maize in upland fields of China were 222 and 197 kg ha^−1^, respectively. The total consumption of synthetic N on wheat and maize was 12.63 Mt year^−1^. At the national scale, the GHG emissions associated with the manufacture of synthetic N fertilizers were estimated to be 41.44 and 59.71 Mt CO_2_-eq year^−1^ for wheat and maize in China, respectively. And the direct N_2_O emissions derived from synthetic N fertilization were estimated to be 35.82 and 69.44 Gg N_2_O year^−1^ for wheat and maize, respectively. In the main wheat-cultivating regions of China, area-scaled GHG emissions were higher for Inner Mongolia, Jiangsu and Xinjiang provinces. And for maize, Gansu, Xinjiang, Yunnan, Shannxi and Jiangsu provinces had higher area-scaled GHG emissions. Higher yield-scaled GHG emissions for wheat and maize mainly occured in Yunnan and Gansu provinces.

**Conclusions:**

The manufacture and application of synthetic N fertilizers for wheat and maize in Chinese croplands is an important source of agricultural GHG emissions. The current study could provide a scientific basis for establishing an inventory of upland GHG emissions in China and developing appropriate mitigation strategies.

## Background

The continuing increase of greenhouse gas (GHG) concentrations in the atmosphere is a worldwide concern. GHG emissions resulting from agricultural production accounted for approximately 11% of the global anthropogenic GHG emissions in 2010 [1, 2]. Recently, the GHG emissions from different types of agricultural activities have been estimated in many studies [3–6]. In China, the application of synthetic nitrogen (N) fertilizers contributed greatly to grain production and food safety. However, under the influence of traditional ideas, as well as lack of proper knowledge and scientific guidance, many farmers apply excessive synthetic N fertilizers to croplands [7, 8]. The rapid increase in the consumption of synthetic N fertilizers and low N use efficiency in Chinese croplands have restricted the sustainable development of agriculture and led to a variety of environmental problems [9]. The manufacture of synthetic N fertilizers, including fossil fuel mining and transportation, ammonia synthesis and conversion of ammonia to various N fertilizer products, is an important source of GHG emissions [10]. In 2005, the GHG emissions from N fertilizer manufacturing were estimated to be 260.4 Tg CO_2_-eq, accounting for 4.3% of the total national GHG emissions [10]. Synthetic N fertilization is considered as one of the most significant factors contributing to anthropogenic N_2_O emissions from agricultural soils [11]. Total direct N_2_O emissions from Chinese croplands were estimated to be 313 Gg N_2_O–N in 2007, and the contribution to N_2_O emissions from croplands by synthetic N fertilizers was 79.4% [12]. Therefore, the consumption of synthetic N fertilizers for crop production is a driver of agricultural GHG emissions.

China is the world’s largest consumer of synthetic N fertilizers [13]. In recent years, growing concerns have been raised on the impacts of synthetic N manufacture and fertilization on GHG emissions in China [10, 14, 15]. Accurate estimates of GHG emissions derived from synthetic N manufacture and fertilization are important for national budgets of anthropogenic sources of GHG emissions and for the development of effective management interventions. Wheat and maize are the main upland crops in China. The cultivation areas of wheat and maize accounted for 25.9% and 41.7%, respectively, of the total cultivation areas of major grain crops in China [16]. In the present research, the GHG emissions associated with the manufacture and application of different synthetic N fertilizers consumed by wheat and maize in China were estimated based on the latest provincial level statistics in mainland China using data mining methods. The application rates of different synthetic N fertilizers for wheat and maize in the main upland crops-cultivating provinces of China were incorporated, making the estimation of GHG emissions driven by the manufacture of synthetic N fertilizers more accurate. And direct N_2_O emissions from synthetic N fertilization for wheat and maize were estimated using crop-specific direct N_2_O emission factors for different agricultural regions of China, which could reduce the uncertainty in estimation of regional direct N_2_O emissions from upland fields.

## Methods

### Data sources

In the current research, thirteen main wheat-cultivating provinces and nineteen main maize-cultivating provinces in mainland China were selected for the study. These provinces account for 96.3% and 96.7%, respectively, of the total cultivation area of wheat and maize in Chinese croplands [16], therefore when we refer to the “national” scale in this paper, we are referring to data for all the selected provinces. The main upland crop-cultivating provinces in the present study can be divided into five agricultural regions: Northeast China (NEC), North China (NC), Middle and lower reaches of Yangtze River (MLYR), Northwest China (NWC), and South and Southwest China (SSWC) (Table 1). To avoid large interannual variations, the GHG emissions from the manufacture and application of synthetic N fertilizers for main upland crops in these provinces of China were estimated based on analysis of latest statistical data available for the period of 2015–2017. The annual cultivation areas and yields of wheat and maize in each province were obtained from National Bureau of Statistics of China [16]. The application rates of different synthetic N fertilizers for wheat and maize in the provincial level were obtained from Department of Price in National Development and Reform Commission of China [17]. In this research, four synthetic N fertilizer products were included: urea, compound fertilizers (N–P_2_O_5_–K_2_O) (CF), diammonium phosphate (DAP), and ammonium bicarbonate (ABC). The N contents of CF were calculated according to the proportion of nutrients (N:P_2_O_5_:K_2_O = 1:1:1) in the most commonly used CF in Chinese croplands [18].

**Table 1:** Regional crop-specific direct N_2_O emission factors for synthetic N fertilizer in Chinese uplands

Agricultural region	Province	Direct N_2_O emission factor (kg N_2_O–N kg N^−1^)	
		Wheat	Maize
NEC	Liaoning, Jilin, Heilongjiang	–	0.0051
NC	Hebei, Shanxi, Shandong, Henan, Inner Mongolia	0.0028	0.0070
MLYR	Jiangsu, Anhui, Hubei	0.0086	0.0067
NWC	Shannxi, Gansu, Xinjiang	0.0032	0.0057
SSWC	Chongqing, Sichuan, Guizhou, Yunnan, Guangxi	0.0050	0.0047

Data are from a study by Yue et al. [21]

*NEC* Northeast China, *NC* North China, *MLYR* Middle and lower reaches of Yangtze River, *NWC* Northwest China, *SSWC* South and Southwest China

### Determination of GHG emissions from synthetic N manufacture

The GHG emission factors along the synthetic N fertilizer chain include fossil fuel mining and transportation, ammonia synthesis and conversion of ammonia to various synthetic N fertilizer products [10]. In the present research, the release of GHG associated with synthetic N manufacture was estimated using localized GHG emission factors for different synthetic N fertilizers in China. Zhang et al. [10] collected parameters specific to China’s N fertilizer manufacture, and they estimated the GHG emission factors for several types of synthetic N fertilizer through a life cycle assessment approach beginning from the mining and transportation of fossil fuel used in the N fertilizer industry to N product manufacturing. In the current work, the GHG emission factors for different types of synthetic N fertilizers, such as 8.1 kg CO_2_-eq kg urea-N^−1^, 7.4 kg CO_2_-eq kg CF-N^−1^ and 7.2 kg CO_2_-eq kg ABC-N^−1^, were adopted from Zhang et al. [10] to evaluate GHG emissions related to the manufacture of synthetic N fertilizers consumed by wheat and maize in Chinese croplands. China’s average-level manufacturing GHG emission factor for DAP was 10.3 kg CO_2_-eq kg DAP-N^−1^ [19]. For each province, the GHG emissions associated with the manufacture of synthetic N fertilizers consumed by wheat and maize were calculated by the following Eq. (1):

1 $${\text{E}}_{\text{M}} = {\text{NR}} \times {\text{A}} \times {\text{EF}}_{\text{M}}$$

where E_M_ represents the release of GHG from the manufacture of different synthetic N fertilizers for wheat or maize in each province (kg CO_2_-eq). NR represents the application rate of urea, CF, DAP and ABC on wheat or maize in each province (kg N ha^−1^). A represents the cultivation area of wheat or maize in each province (ha). EF_M_ represents the GHG emission factor for the manufacture of urea, CF, DAP or ABC in China (kg CO_2_-eq kg N^−1^).

### Determination of direct N_2_O emissions from synthetic N fertilization

In the current research, the direct N_2_O emissions from synthetic N fertilization for wheat and maize in China were estimated using IPCC Guidelines for National Greenhouse Gas Inventories [20] combined with agroregion- and crop-specific direct N_2_O emission factors for upland fields of China from a research by Yue et al. [21] (Table 1). Therefore, Eq. (2) was used to estimate the direct N_2_O emissions from upland fields of China due to synthetic N fertilization:

2 $${\text{E}}_{\text{D}} = {\text{NR}} \times {\text{A}} \times {\text{EF}}_{\text{D}} \times 4 4/ 2 8$$

where E_D_ represents the direct N_2_O emissions from synthetic N fertilization for wheat or maize in each province (kg N_2_O), where EF_D_ represents the direct N_2_O emission factor for synthetic N applied to wheat or maize in each agricultural region (kg N_2_O–N kg N^−1^), and the fraction 44/28 converts N to N_2_O.

Area-scaled (kg CO_2_-eq ha^−1^) and yield-scaled (kg CO_2_-eq kg grain^−1^) GHG emissions from synthetic N manufacture and fertilization for wheat and maize in different provinces of China were estimated according to Eqs. (3) and (4), respectively:

3 $${\text{Area-scaled GHG emissions}} = [\left( {{\text{E}}_{\text{M}} + {\text{ E}}_{\text{D}} } \right) \times 2 9 8]/{\text{A}}$$

4 $${\text{Yield-scaled GHG emissions}} = \left[ {\left( {{\text{E}}_{\text{M}} + {\text{E}}_{\text{D}} } \right) \times 2 9 8} \right]/{\text{Y}}$$

where Y represents the yields of wheat or maize in each province of China (kg), and the constant 298 is the radiative forcing constant of N_2_O relative to CO_2_ at a 100-year time horizon [22].

## Results

### GHG emissions from synthetic N manufacture for wheat and maize in China

**Fig. 1:**
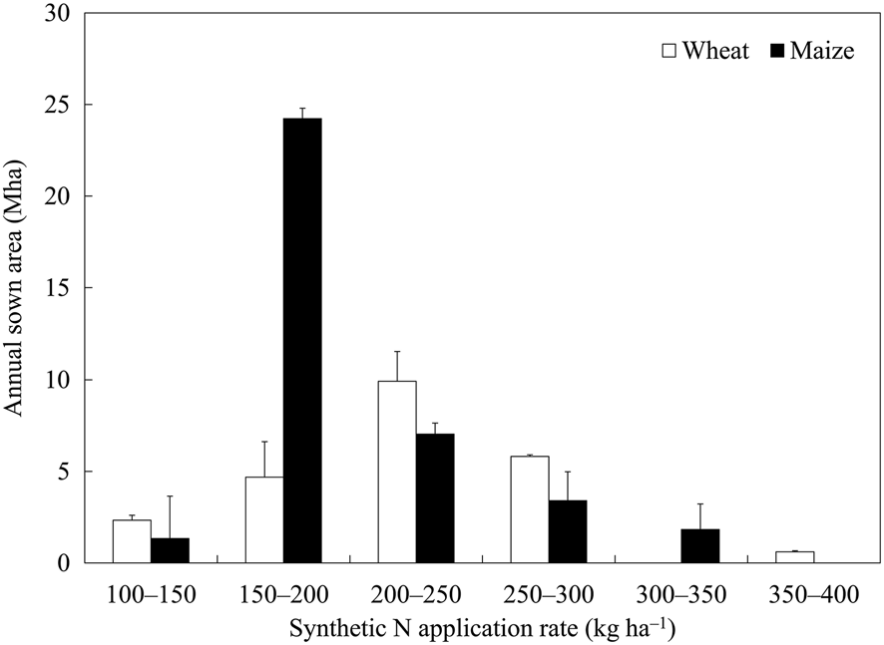
Status of synthetic N fertilization for wheat and maize in China (2015–2017). Error bars in the figure represent standard deviation

In Chinese croplands, the average application rates of synthetic N for wheat and maize were 222 and 197 kg ha^−1^, respectively. At the national scale, the application rates of urea, CF, DAP and ABC for wheat were 124, 72, 17 and 9 kg N ha^−1^, respectively. The application rates of urea, CF, DAP and ABC for maize were 111, 62, 15 and 8 kg N ha^−1^, respectively. In upland fields of China, the area of wheat with synthetic N application rate of 200–250 kg ha^−1^ was the largest, accounting for 42.5% of the total wheat-cultivating areas. The maize-cultivating soils that received 150–200 kg N ha^−1^ covered the largest area, accounting for 64.0% of the total maize-cultivating areas (Fig. 1). At the national level, 5.18 and 7.45 Mt synthetic N fertilizers were consumed annually for wheat and maize, respectively.

**Fig. 2:**
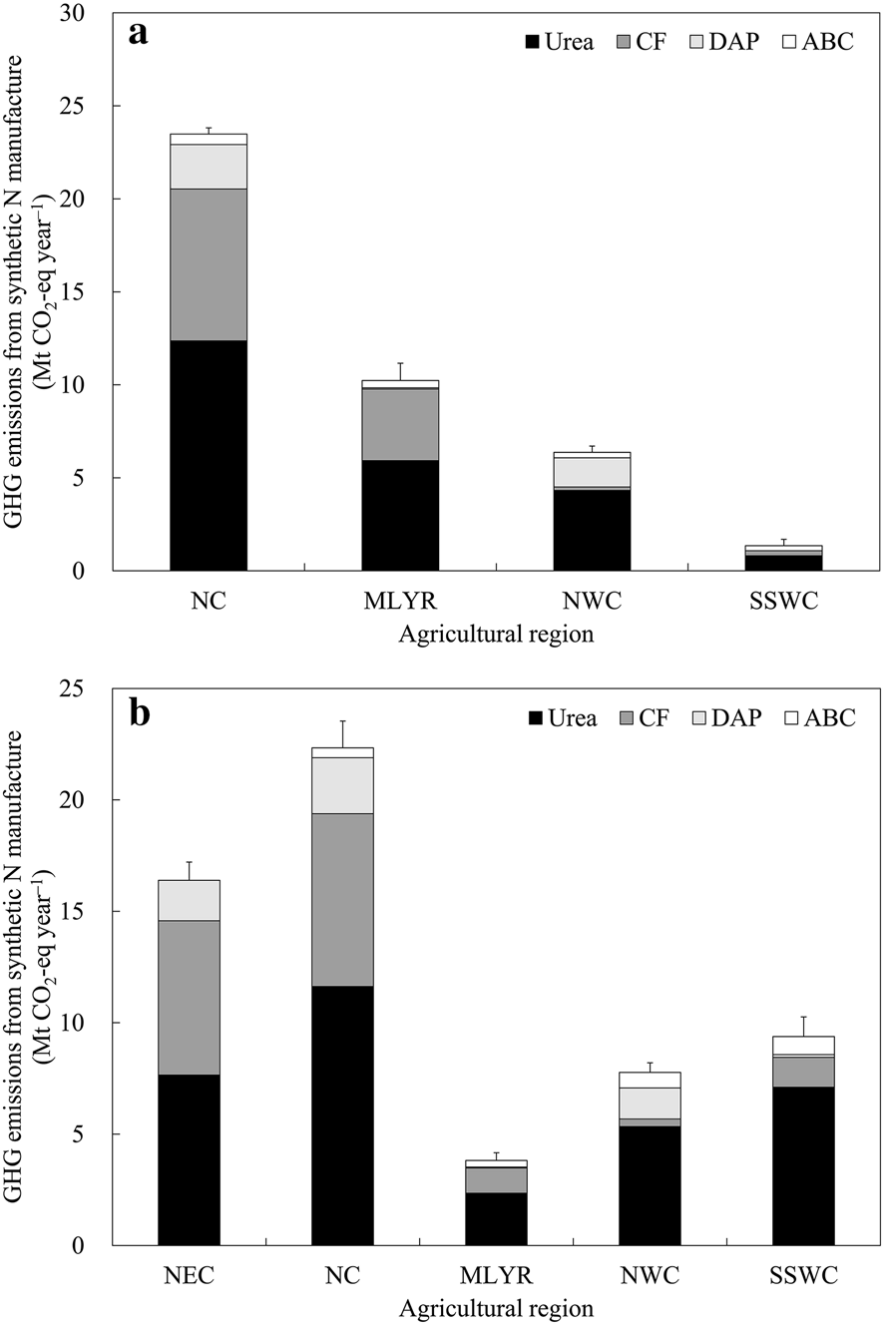
Estimated average annual greenhouse gas (GHG) emissions from synthetic N manufacture for wheat (**a**) and maize (**b**) in different agricultural regions of China (2015–2017). Error bars in the figure represent standard deviation. *CF* compound fertilizers, *DAP* diammonium phosphate, *ABC* ammonium bicarbonate, *NEC* Northeast China, *NC* North China, *MLYR* Middle and lower reaches of Yangtze River, *NWC* Northwest China, *SSWC* South and Southwest China

Considering the cultivation areas and application rates of different synthetic N fertilizers for wheat and maize in each province, the GHG emissions associated with the manufacture of synthetic N fertilizers consumed by wheat and maize in Chinese croplands were estimated using Eq. (1). As shown in Table 2, in the main wheat-cultivating areas of China, the estimated emissions of GHG from synthetic N manufacture were higher from Henan, Shandong, Hebei, Jiangsu and Anhui provinces, accounting for 21.1%, 16.0%, 12.1%, 11.7% and 10.0% of the total GHG emissions from the selected provinces, respectively. In the main maize-cultivating areas, the GHG emissions associated with the manufacture of synthetic N fertilizers were mainly released in Heilongjiang, Inner Mongolia, Jilin, Shandong, Hebei, Henan and Yunnan provinces, accounting for 13.3%, 10.6%, 8.6%, 8.6%, 7.1%, 6.7% and 6.4% of the total GHG emissions from the selected provinces, respectively. Of the total GHG emissions from the manufacture of synthetic N fertilizers consumed by wheat and maize, approximately 49.4% was estimated to be released in Henan, Shandong, Hebei, Inner Mongolia and Heilongjiang provinces. For wheat, the estimated GHG emissions associated with synthetic N manufacture of NC, MLYR, NWC and SSWC were estimated to be 23.49, 10.23, 6.37 and 1.35 Mt CO_2_-eq year^−1^, respectively (Fig. 2). For maize, the estimated GHG emissions from synthetic N manufacture for NC, NEC, SSWC, NWC and MLYR were 22.34, 16.39, 9.38, 7.77 and 3.82 Mt CO_2_-eq year^−1^, respectively.

**Table 2:** Estimated average annual greenhouse gas (GHG) emissions from synthetic N manufacture for wheat and maize in different provinces of China (2015–2017)

Agricultural region	Province	GHG emissions from synthetic N manufacture (Mt CO_2_-eq year^−1^)	
		Wheat	Maize
NEC	Liaoning	–	3.32 ± 0.09
	Jilin	–	5.14 ± 0.10
	Heilongjiang	–	7.94 ± 0.68
NC	Hebei	5.01 ± 0.12	4.23 ± 0.22
	Shanxi	1.16 ± 0.12	2.65 ± 0.14
	Shandong	6.65 ± 0.10	5.12 ± 0.29
	Henan	8.74 ± 0.20	3.99 ± 0.06
	Inner Mongolia	1.94 ± 0.19	6.35 ± 0.59
MLYR	Jiangsu	4.86 ± 0.38	1.02 ± 0.12
	Anhui	4.14 ± 0.56	1.61 ± 0.20
	Hubei	1.22 ± 0.01	1.19 ± 0.05
NWC	Shaanxi	2.03 ± 0.20	2.64 ± 0.17
	Gansu	1.36 ± 0.07	2.64 ± 0.17
	Xinjiang	2.98 ± 0.18	2.49 ± 0.12
SSWC	Chongqing	–	0.75 ± 0.04
	Sichuan	0.86 ± 0.26	2.14 ± 0.34
	Guizhou	–	1.62 ± 0.30
	Yunnan	0.49 ± 0.09	3.84 ± 0.36
	Guangxi	–	1.04 ± 0.06

Values in the table represent mean ± standard deviation

*NEC* Northeast China, *NC* North China, *MLYR* Middle and lower reaches of Yangtze River, *NWC* Northwest China, *SSWC* South and Southwest China

### Direct N_2_O emissions resulting from synthetic N fertilization for wheat and maize in China

**Fig. 3:**
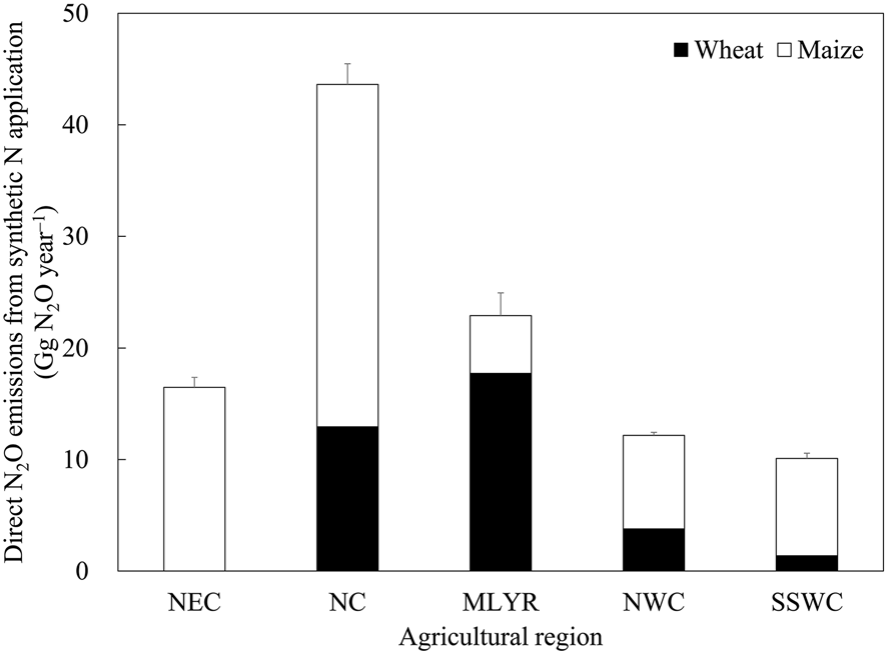
Estimated average annual direct N_2_O emissions from synthetic N fertilization for wheat and maize in different agricultural regions of China (2015–2017). Error bars in the figure represent standard deviation. *NEC* Northeast China, *NC* North China, *MLYR* Middle and lower reaches of Yangtze River, *NWC* Northwest China, *SSWC* South and Southwest China

In fertilized croplands, the GHG emissions due to synthetic N fertilization are mainly in the form of N_2_O. Using Eq. (2), the direct N_2_O emissions resulting from synthetic N fertilizers applied to upland fields of China were estimated (Table 3). In the main wheat-cultivating areas of China, Jiangsu, Anhui, Henan, Shandong and Hebei provinces were the major sources of direct N_2_O emissions. And the synthetic N-induced direct emissions of N_2_O for these provinces accounted for 23.2%, 20.2%, 13.7%, 10.3% and 7.6%, respectively, of the total direct N_2_O emissions from synthetic N fertilization in the main wheat-cultivating regions. In the main maize-cultivating provinces of China, direct emissions of N_2_O were higher from Inner Mongolia, Heilongjiang, Shandong, Hebei, Henan and Jilin provinces. The synthetic N-induced direct N_2_O emissions of these provinces accounted for 12.1%, 11.2%, 10.3%, 8.3%, 8.2% and 7.7%, respectively, of the total direct N_2_O emissions from synthetic N fertilization for the main maize-cultivating areas of China. In the main wheat-cultivating regions of China, the direct N_2_O emissions associated with synthetic N fertilization of MLYR, NC, NWC and SSWC were 17.73, 12.94, 3.78 and 1.37 Gg N_2_O year^−1^, respectively (Fig. 3). And for maize, the direct N_2_O emissions from synthetic N fertilization for NC, NEC, SSWC, NWC and MLYR were 30.68, 16.48, 8.74, 8.40 and 5.14 Gg N_2_O year^−1^, respectively. At the national scale, the total direct N_2_O emissions associated with synthetic N fertilization were estimated to be 105.26 Gg N_2_O year^−1^ for main upland crops in China.

**Table 3:** Estimated average annual direct N_2_O emissions from synthetic N fertilization for wheat and maize in different provinces of China (2015–2017)

Agricultural region	Province	Direct N_2_O emissions (Gg N_2_O year^−1^)	
		Wheat	Maize
NEC	Liaoning	–	3.38 ± 0.10
	Jilin	–	5.32 ± 0.16
	Heilongjiang	–	7.78 ± 0.66
NC	Hebei	2.71 ± 0.07	5.78 ± 0.27
	Shanxi	0.63 ± 0.07	3.65 ± 0.20
	Shandong	3.68 ± 0.05	7.12 ± 0.44
	Henan	4.92 ± 0.10	5.71 ± 0.12
	Inner Mongolia	1.01 ± 0.10	8.42 ± 0.78
MLYR	Jiangsu	8.32 ± 0.64	1.34 ± 0.14
	Anhui	7.22 ± 0.96	2.17 ± 0.27
	Hubei	2.20 ± 0.01	1.63 ± 0.06
NWC	Shaanxi	1.22 ± 0.13	2.96 ± 0.19
	Gansu	0.82 ± 0.04	2.85 ± 0.18
	Xinjiang	1.74 ± 0.10	2.59 ± 0.12
SSWC	Chongqing	–	0.71 ± 0.04
	Sichuan	0.89 ± 0.27	2.06 ± 0.32
	Guizhou	–	1.50 ± 0.28
	Yunnan	0.48 ± 0.09	3.50 ± 0.32
	Guangxi	–	0.97 ± 0.06

Values in the table represent mean ± standard deviation

*NEC* Northeast China, *NC* North China, *MLYR* Middle and lower reaches of Yangtze River, *NWC* Northwest China, *SSWC* South and Southwest China

### Area- and yield-scaled GHG emissions from synthetic N manufacture and fertilization for wheat and maize in China

**Fig. 4:**
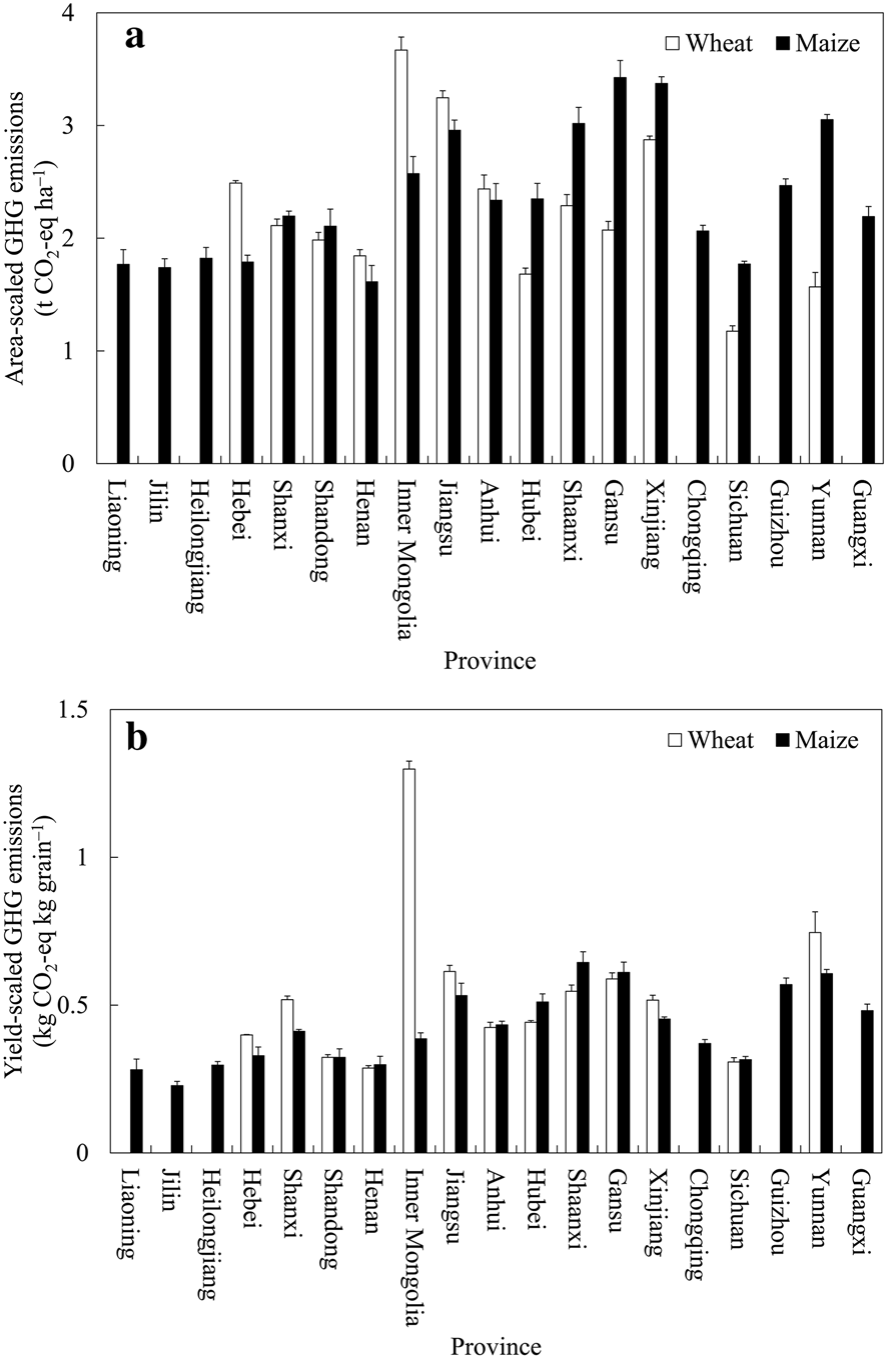
Area- (**a**) and yield-scaled (**b**) greenhouse gas (GHG) emissions from synthetic N manufacture and fertilization for wheat and maize in different provinces of China (2015–2017). Error bars in the figure represent standard deviation

At the national scale, the GHG emissions from synthetic N manufacture and fertilization were estimated to be 52.11 and 80.40 Mt CO_2_-eq year^−1^ for wheat and maize, respectively (Table 4). For wheat, the GHG emissions derived from synthetic N manufacture and fertilization were 41.44 and 10.67 Mt CO_2_-eq year^−1^, respectively. For maize, the GHG emissions derived from synthetic N manufacture and fertilization were 59.71 and 20.69 Mt CO_2_-eq year^−1^, respectively. By substituting the GHG emissions resulting from synthetic N manufacture and fertilization for wheat and maize in each province, as well as the cultivation areas and yields of wheat and maize, into Eqs. (3) and (4), area- and yield-scaled GHG emissions for wheat and maize in different provinces of China were estimated (Fig. 4). In the main wheat-cultivating areas of China, area-scaled GHG emissions were higher from Inner Mongolia, Jiangsu and Xinjiang provinces, which were 3.67, 3.25 and 2.87 t CO_2_-eq ha^−1^, respectively (Fig. 4a). In the main maize-cultivating areas of China, Gansu, Xinjiang, Yunnan, Shannxi and Jiangsu had higher area-scaled GHG emissions of 3.43, 3.37, 3.05, 3.02 and 2.96 t CO_2_-eq ha^−1^, respectively. In the main wheat-cultivating areas of China, higher yield-scaled GHG emissions were mainly distributed in Inner Mongolia, Yunnan, Jiangsu and Gansu provinces, which were 1.30, 0.75, 0.61 and 0.59 kg CO_2_-eq kg grain^−1^, respectively (Fig. 4b). And in the main maize-cultivating areas, yield-scaled GHG emissions in Shaanxi, Gansu, Yunnan and Guizhou were estimated to be higher, which were 0.64, 0.61, 0.61 and 0.57 kg CO_2_-eq kg grain^−1^, respectively. At the regional scale, higher area- and yield-scaled GHG emissions for upland crops were mainly distributed in NWC and MLYR (Table 5). At the national level, area-scaled GHG emissions from synthetic N manufacture and fertilization for wheat and maize were 2.23 and 2.13 t CO_2_-eq ha^−1^, respectively. And yield-scaled GHG emissions for wheat and maize were 0.41 and 0.35 kg CO_2_-eq kg grain^−1^, respectively.

**Table 4:** Estimated average annual greenhouse gas (GHG) emissions from synthetic N manufacture and fertilization for wheat and maize in China (2015–2017)

	Wheat	Maize
GHG emissions from urea manufacture (Mt CO_2_-eq year^−1^)	23.42 ± 0.19	34.07 ± 1.00
GHG emissions from CF manufacture (Mt CO_2_-eq year^−1^)	12.47 ± 0.70	17.49 ± 2.34
GHG emissions from DAP manufacture (Mt CO_2_-eq year^−1^)	4.03 ± 0.13	5.89 ± 0.34
GHG emissions from ABC manufacture (Mt CO_2_-eq year^−1^)	1.53 ± 0.43	2.25 ± 0.12
Direct N_2_O emissions from the soil (Gg N_2_O year^−1^)	35.82 ± 1.20	69.44 ± 4.26
Total national GHG emissions (Mt CO_2_-eq year^−1^)	52.11 ± 0.93	80.40 ± 4.88

Values in the table represent mean ± standard deviation

*CF* compound fertilizers, *DAP* diammonium phosphate, *ABC* ammonium bicarbonate

**Table 5:** Area- and yield-scaled greenhouse gas (GHG) emissions from synthetic N manufacture and fertilization for wheat and maize in different agricultural regions of China (2015–2017)

Agricultural region	Area-scaled GHG emissions (t CO_2_-eq ha^−1^)		Yield-scaled GHG emissions (kg CO_2_-eq kg grain^−1^)	
	Wheat	Maize	Wheat	Maize
NEC	–	1.78 ± 0.04	–	0.27 ± 0.01
NC	2.10 ± 0.03	2.04 ± 0.07	0.35 ± 0.01	0.34 ± 0.02
MLYR	2.60 ± 0.07	2.48 ± 0.10	0.50 ± 0.01	0.48 ± 0.02
NWC	2.47 ± 0.03	3.26 ± 0.10	0.54 ± 0.01	0.56 ± 0.02
SSWC	1.29 ± 0.06	2.37 ± 0.03	0.39 ± 0.02	0.47 ± 0.01

Values in the table represent mean ± standard deviation

*NEC* Northeast China, *NC* North China, *MLYR* Middle and lower reaches of Yangtze River, *NWC* Northwest China, *SSWC* South and Southwest China

## Discussion

Synthetic N fertilizers use-related GHG emissions accounted for 55% of total emissions from Chinese agriculture [6]. And synthetic N fertilization-induced direct N_2_O emissions represented the largest contribution to total N_2_O emissions from agricultural soils [12]. The current research deals with quantification of GHG emissions from the manufacture of synthetic N fertilizers and direct N_2_O emissions from synthetic N fertilization for wheat and maize of the main upland crops-cultivating areas in China using localized emission factors. In the present study, the annual cultivation areas and yields of wheat and maize in each province were obtained from National Bureau of Statistics of China [16]. And the application rates of different synthetic N fertilizers (urea, CF, DAP and ABC) for wheat and maize in the provincial level were obtained from Department of Price in National Development and Reform Commission of China [17]. For the purpose of reducing temporal variations, the annual cultivation areas of upland crops and the application rates of different synthetic N fertilizers in each province over the three-year period (2015–2017) were averaged based on the latest statistics [16]. By combining survey data of 230 N fertilizer plants (40% of the total N fertilizer industry in China) from Chinese Nitrogen Fertilizer Industry Association and Chinese-specific parameters from published studies, Zhang et al. [10] estimated the GHG emission factors for the manufacture of urea, CF and ABC in China. In the current work, the GHG emission factors from Zhang et al. [10] (8.1 kg CO_2_-eq kg urea-N^−1^, 7.4 kg CO_2_-eq kg CF-N^−1^ and 7.2 kg CO_2_-eq kg ABC-N^−1^) and Chen et al. [19] (10.3 kg CO_2_-eq kg DAP-N^−1^) were adopted to evaluate the release of GHG related to the manufacture of different synthetic N fertilizers consumed in upland fields of China.

N fertilizer-induced direct N_2_O emissions from agricultural fields are critically affected by many factors, including N application rate, climate variables, crop types and soil characteristics [21, 23–26]. Several models have been developed and used to estimate N fertilizer-induced N_2_O emissions from the soil [25, 27, 28]. Due to limited availability of enough relevant information, the determination of direct N_2_O emissions from synthetic N fertilization for wheat and maize in China in the current work was done using the IPCC emission factor method combined with region- and crop-specific direct N_2_O emission factors. The IPCC method is a universally accepted method for calculating the national inventory of direct N_2_O emissions from the application of synthetic N fertilizers, and it is easy to make comparisons among countries [12]. This method has been applied in some previous studies to estimate synthetic N fertilizer-induced direct N_2_O emissions from Chinese croplands [10, 12, 21, 23]. The study of Lu et al. [29] suggested that crop-specific direct N_2_O emission factor for upland crops in China was 0.0074 kg N_2_O-N kg N^−1^. The direct N_2_O emission factor for Chinese uplands due to synthetic N fertilization was estimated to be 0.0105 kg N_2_O-N kg N^−1^ by Gao et al. [12], based on 261 N_2_O emission measurements in upland fields. The results of Zhou et al. [25] indicated that N fertilizer-induced emission factor of N_2_O was 0.0084 kg N_2_O–N kg N^−1^ for Chinese upland grain crops by considering various predictors. China has a large agricultural area with diverse climate types. Regional heterogeneity in synthetic N-induced N_2_O emission factor should be considered when assessing the direct emissions of N_2_O from N fertilizer consumption. Regional and crop-specific N_2_O emission factors were proposed by Aliyu et al. [23] based on 151 synthetic N-induced N_2_O emission factors from agricultural fields across China. To minimize the uncertainty in estimates of synthetic N-induced direct N_2_O emissions from Chinese uplands, latest agroregion- and crop-specific N_2_O emission factors from a study by Yue et al. [21] based on a database of 1151 field observations across China were used in this research. The applied N-induced N_2_O emissions from Chinese fertilized uplands were quantified to be 180 Gg N_2_O-N in 1997, using precipitation-rectified N_2_O emission factor [29]. In 2007, direct N_2_O emissions from synthetic N fertilization in Chinese upland fields were calculated to be 224 Gg N_2_O-N [12]. The direct N_2_O emissions from synthetic N application for wheat and maize under 2016 cultivation were estimated to be 116 Gg N_2_O-N using the regional N_2_O emission factors of N applied [23]. In this study, the total direct N_2_O emissions associated with synthetic N fertilization were estimated to be 105 Gg N_2_O year^−1^ for wheat and maize in China during the period of 2015–2017. Compared with previous studies, the calibrated direct N_2_O emission factors in the study of Yue et al. [21] had the relatively low uncertainty. Therefore, the adoption of crop-specific direct N_2_O emission factors at the regional level from Yue et al. [21] would reduce uncertainty in the estimation of direct N_2_O emissions derived from synthetic N fertilization in Chinese uplands. The survey on the consumption of fossil fuel energy, electricity use and total energy consumption of 230 N fertilizer plants in China was conducted between 2002 and 2005 [10]. There is still an issue of uncertainty for the estimation of GHG emissions from synthetic N manufacture in this study. Nevertheless, the results of this work may offer fundamental information for establishing inventory of GHG emissions from Chinese uplands and identifying effective mitigation options.

Synthetic N fertilizer is commonly overused in the intensive cereal cropping systems of China [7, 8]. During the 2015–2017 period, the average application rates of synthetic N for wheat and maize in upland fields of China were estimated to be 222 and 197 kg ha^−1^, respectively. The results of this study indicated that the area of synthetic N application rates higher than 200 kg ha^−1^ accounted for 70.0% in wheat and 32.4% in maize. The over-application of synthetic N fertilizers is an important source of GHG emissions. The order of contributions to GHG emissions related to synthetic N manufacture for wheat and maize in Chinese croplands was urea > CF > DAP > ABC. Area-scaled GHG emissions for wheat were higher from Inner Mongolia, Jiangsu and Xinjiang provinces. For maize, Gansu, Xinjiang, Yunnan, Shannxi and Jiangsu provinces had relatively high area-scaled GHG emissions. There is a greater potential for GHG mitigation in the above provinces. More corrective actions should be undertaken in these priority areas.

China plays an important role in the global N cycle, due to its role as the world’s largest consumer of synthetic N fertilizers [13]. The manufacture and application of synthetic N fertilizers is recognized as a significant factor contributing to GHG emissions from crop production. Therefore, the management of synthetic N fertilization in Chinese croplands has caused extensive concern. Reduction of synthetic N usage can decrease the amount of synthetic N fertilizer demand and cut GHG release from synthetic N manufacture and fertilization. For the purpose of mitigating emissions of GHG from Chinese agriculture without sacrificing crop yields, optimizing synthetic N application rate to a reasonable level in over-fertilized areas is an important and essential strategy. Relevant research has indicated that reducing synthetic N application rate in Chinese croplands is feasible by adopting sustainable nutrient management techniques, such as Nutrient Expert for fertilizer recommendation decision support system and in-season N management strategy [30–32]. It is the consensus of many experts that N use efficiency in Chinese croplands can be improved through: deep placement of N fertilizer; maximizing the competitive absorptive capacity of crop root system by eliminating constraints affecting crop growth, including balanced fertilization, integrated management of water and fertilizer, and applying N fertilizer at the period when the root system has strong absorption capacity [33, 34]. In China, promoting a wider adoption of better N management practices is crucial for mitigating GHG emissions associated with synthetic N fertilization while maintaining high grain yields. Replacement of conventional synthetic N fertilizers by enhanced efficiency fertilizers (i.e., control release fertilizers, nitrification inhibitors and urease inhibitors) could help to reduce direct N_2_O emissions from the soil [21, 35, 36]. And reducing synthetic N input coupled with manure N incorporation is also an efficient practice for mitigating N_2_O emissions without compromising productivity [37, 38].

Proper application of synthetic N demands a combination of scientific guidance, economic incentives and policy interventions. The findings of Huang et al. [39] indicated that delivering appropriate information and knowledge on N fertilizer use to farmers was effective in reducing overall N fertilizer use by 22% in maize production in the North China Plain, which has important policy implications. The Ministry of Agriculture and Rural Affairs of China should take effective measures, including carrying out agricultural technology extension activities, providing feasible advice to farmers in different agricultural regions, and formulating crop and area-specific fertilization norms [40]. The Chinese agricultural sector must improve the performance of the agricultural extension service and incentivize small-holder farmers to gradually adopt optimum N fertilization techniques. Compared with curriculum training, field guidance in the form of follow-ups offered by agricultural technicians is more useful for improving farmers’ knowledge of fertilizer management [41]. In China, Soil Testing and Fertilizer Recommendation Program has been implemented across the nation to reduce synthetic N use and enhance yields of main grain crops since 2005. In 2013, the Ministry of Agriculture of the People’s Republic of China released the recommendation for soil testing-based mineral fertilizer application for maize, wheat and rice. In 2015, the Ministry of Agriculture of China announced the Action Plan to Achieve Zero-growth of Synthetic Fertilizer Application by 2020. China is now on the right track to implement improved N management techniques in croplands, which would help to identify options for lowering GHG emissions from agricultural production. In recent years, N_2_O emissions from Chinese croplands have grown slowly, owing to nationwide policy interventions and prevalence of technological adoptions [28, 42, 43]. Moreover, technological innovation in China’s N fertilizer industry could make a significant contribution to synthetic N manufacture-related GHG emissions reduction [10]. Adopting more advanced technologies for the N fertilizer chain is essential for reducing GHG emissions resulting from the manufacture of synthetic N fertilizers used in Chinese wheat and maize production systems. Utilizing appropriate type of N fertilizer is also a relatively effective means of ameliorating synthetic N manufacture-induced GHG emissions [14]. In comparison with small household farms, aggregated farms had lower GHG emissions due to N fertilizer application [44]. Therefore, developing aggregated farm scale with improved management in land consolidation programs would provide a great opportunity for agricultural GHG emissions mitigation in China.

## Conclusions

The manufacture and application of synthetic N fertilizers for raising wheat and maize in Chinese croplands is an important driver of agricultural GHG emissions. Nationwide and over a three-year period (2015–2017), the average annual total application of synthetic N to wheat- and maize-cultivating soils was 5.18 and 7.45 Mt year^−1^, respectively. The average annual total national emissions of GHG related to synthetic N manufacture and fertilization were estimated to be 52.11 and 80.40 Mt CO_2_-eq year^−1^ for wheat and maize in China, respectively. In the main wheat-cultivating areas of China, area-scaled GHG emissions were higher for Inner Mongolia, Jiangsu and Xinjiang provinces. In the main maize-cultivating areas, Gansu, Xinjiang, Yunnan, Shannxi and Jiangsu had higher area-scaled GHG emissions. Higher yield-scaled GHG emissions for wheat and maize in China mainly occurred in Yunnan and Gansu provinces. The results is useful in establishing an inventory of Chinese uplands GHG emissions and identifying key mitigation options.

## Data Availability

The datasets used in this article are available upon request.

## Abbreviations

N: nitrogen
GHG: greenhouse gas
CO_2_-eq: CO_2_-equivalent
CF: compound fertilizers
DAP: diammonium phosphate
ABC: ammonium bicarbonate
NEC: Northeast China
NC: North China
MLYR: Middle and lower reaches of Yangtze River
NWC: Northwest China
SSWC: South and Southwest China
